# Reversibility of glioma stem cells’ phenotypes explains their complex *in vitro* and *in vivo* behavior: Discovery of a novel neurosphere-specific enzyme, cGMP-dependent protein kinase 1, using the genomic landscape of human glioma stem cells as a discovery tool

**DOI:** 10.18632/oncotarget.11589

**Published:** 2016-08-24

**Authors:** Thomas J. Wilson, Daniel B. Zamler, Robert Doherty, Maria G. Castro, Pedro R. Lowenstein

**Affiliations:** ^1^ Department of Neurosurgery, The University of Michigan School of Medicine, Ann Arbor, MI, USA; ^2^ Department of Cell and Developmental Biology, Training Programs in Cancer Biology, Immunology & Neurosciences, The University of Michigan School of Medicine, Ann Arbor, MI, USA

**Keywords:** glioblastoma, stem cells, plasticity, PRKG1, genomics

## Abstract

Glioma cells grow in two phenotypic forms, as adherent monolayers and as free floating “neurospheres/tumorspheres”, using specific media supplements. Whether each phenotype is irreversible remains unknown. Herein we show that both states are reversible using patient derived glioblastoma cell cultures (i.e., HF2303, IN859, MGG8, IN2045). Both phenotypic states differ in proliferation rate, invasion, migration, chemotaxis and chemosensitivity. We used microarrays to characterize gene expression across the patient derived glioblastoma cell cultures, to find specific inhibitors of the sphere population. Traditional chemotherapeutics (i.e., doxorubicin or paclitaxel) inhibit rapidly dividing adherent cells; it has been more challenging to inhibit the growth of the sphere phenotype. PRKG1, known to induce apoptosis when activated, is increased in all patient derived glioblastoma spheres. Stimulation of PRKG1 activity preferentially reduced cell viability in the sphere phenotype. Computational network and gene ontology analysis identified novel potential target genes linked to the PRKG1 expression node.

## INTRODUCTION

Lack of a precise experimental definition of glioma stem cells has led to variance in the way these cells are cultured *in vitro*, characterized in *in vitro* assays, and to confusing nomenclature. A conceptual and practical definition of glioma stem cells exists and characterizes these cells by their ability to form tumors *in vivo* following implantation, extensive self-renewal, asymmetric division generating tumorigenic and non-tumorigenic cells, multilineage differentiation potential, and formation of neuro/tumorspheres *in vitro*. An exact experimental definition has been in flux. In 2003, Singh et al., [3] published the first identification and purification of human brain tumor stem cells. They defined human brain tumor stem cells as being capable of forming neurospheres in serum-free medium, expressing markers of differentiation when grown adherently on L-poly-ornithine coated coverslips in fetal bovine serum supplemented medium, and being CD133+ [3]. Subsequent to this report, the definition was expanded to include tumor growth when transplanted into rodent models [4,5]. This experimental definition was challenged when CD133- cells were shown to form tumor spheres *in-vitro* and tumors *in-vivo* [6–8]. So far no markers or phenotypic functions have been shown to be unequivocally pathognomonic of glioma stem cells, although a number have been tried and reported, including immunophenotyping for markers other than CD133, side population analysis, aptamer selection, and intrinsic autofluorescence [9–14]. Despite conflicting evidence, methods’ sections of many, yet not all papers, refer to CD133+ as the defining marker of glioma stem cells [15]. Equally CD133 expression continues to be the cornerstone definition of glioma stem cells by some groups [11]. Nevertheless, given contradictory evidence for CD133+ cells being the pathognomonic marker of glioma stem cells, there does not appear to exist one single universally accepted definition of glioma stem cells. In our experiments, as CD133 was expressed by cells of both the sphere and adherent phenotype, it could not be used as a differential marker.

Inconsistencies continue to abound in the literature. In many studies reporting on glioma stem cells, brain tumor propagating cells, brain tumor initiating cells, etc., it remains unclear which definition is being used. This makes replication, interpretation and generalization of these studies difficult. Some studies describe glioma stem cells in *in vitro* cultures as adherent monolayers [16–18], while others only accept neurosphere-like tumorspheres [3, 18, 19]. The significance of varied methods of culture remains to be clarified. For example, whether cells behave differently under these two culture conditions and whether the method of culture modifies the outcome of assays such as the testing of chemotherapeutics deserves attention. Equally, the identification of the sub-population of cells that function as stem cells *in vivo* remains unsolved. It is possible that clarification of the *in vitro* work will help address the *in vivo* challenges as well.

In response to these challenges, we propose a simple, reproducible experimental definition of glioma propagating/initiating cells (GPCs). To create an experimental definition of GPCs and a new method for the identification of potential therapeutic molecules, we focused on understanding the ramifications of the variance of the culture conditions. We hypothesized that GPCs would grow both as an adherent monolayer and as neurospheres but that their behavior would depend on phenotype and culture conditions, i.e., that the glioma stem cell state is dynamic and allows reversible switching between both states. We further hypothesized that comparing genetic expression profiles would inform us about the signaling mechanisms responsible any observed differences in proliferation, invasion, and chemoresistance. We tested these hypotheses, and found that all patient derived glioblastoma cell cultures tested can grow reversibly as an adherent monolayer and as tumorspheres. Each culture condition/phenotype, however, has different characteristics. For example, the adherent phenotype was characterized by higher proliferation, higher invasion, and lower resistance to chemotherapy *in vitro*. We wanted to utilize comparative genetic expression profiles in order to reveal potential therapeutic molecules in the chemoresistant sphere phenotype thought to cause recurrence of gliomas WHO IV. By identifying such molecules in the sphere population we hope to inhibit both the adherent proliferative population as well as the quiescent chemo-resistant sphere phenotype. cGMP-protein dependent kinase I (PRKG1) increases in spheres. This effect occurred in a cell-cycle independent fashion and was observed most notably in the subset of cells that were highly resistant to traditional chemotherapy. Thus, PRKG1 represents a novel therapeutic target to inhibit growth of glioma stem cells.

## RESULTS

### The adherent phenotype shows a higher proliferation rate

**Figure 1:**
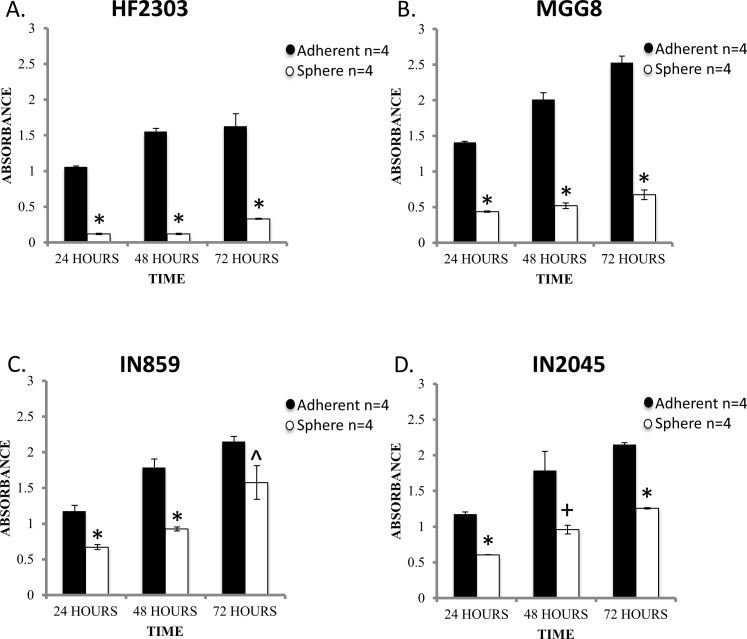
Within each patient derived glioblastoma cell culture, the adherent phenotype is characterized by a higher rate of proliferation compared to sphere phenotype as assessed by MTS assay This finding was consistent across the 24, 48, and 72 hour time points. *n* = 4 for all conditions. ^ *p* < 0.05, + *p* < 0.01, *p < 0.001.

To test our hypotheses we sought to determine if the distinct growth patterns confer different behavioral patterns. We compared the proliferation rate of cells under the two conditions by MTS assay. Cells grown adherently demonstrated a higher proliferation rate than the same cells grown under sphere conditions for all four patient derived glioblastoma cell cultures tested (Figure 1). CD133 was expressed by cells of both the sphere and adherent phenotype (Figure S1), which made it unsuitable to use as a differential marker for glioma stem cells.

### Adherent phenotype shows higher migration and invasion

**Figure 2:**
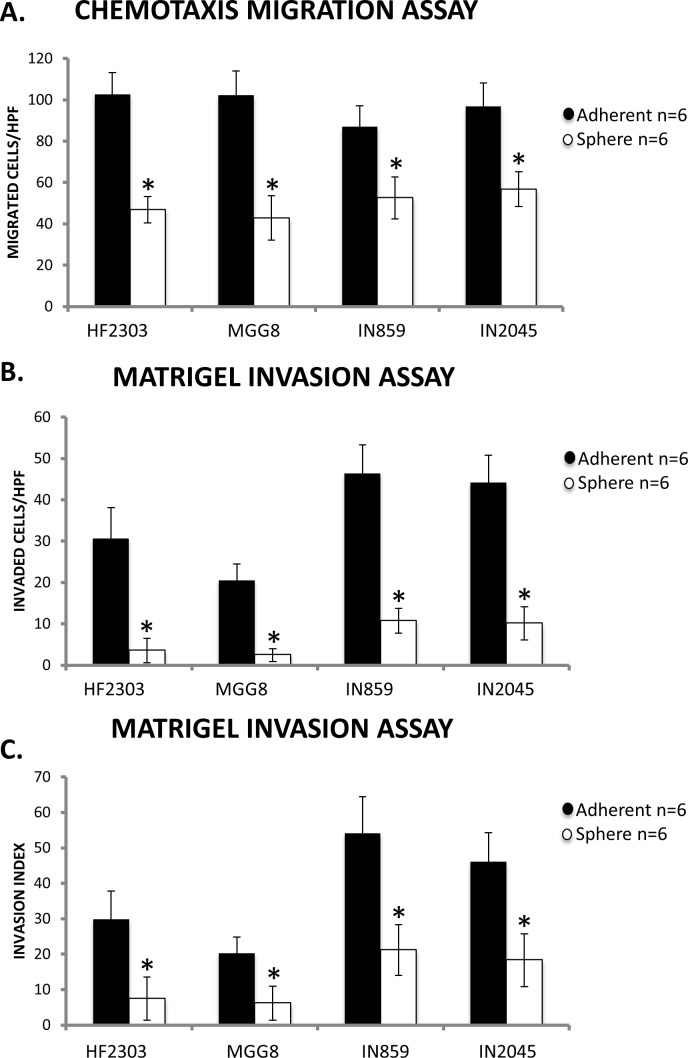
**A.** Within each patient derived glioblastoma cell culture, the adherent phenotype had more migrated cells across a control, non-coated insert compared to the sphere phenotype in a transwell migration assay. **B.** The adherent phenotype also demonstrated a higher number cells invading through a Matrigel matrix compared to the sphere phenotype. **C.** When invasion was normalized to migration to calculate an invasion index, the adherent phenotype still demonstrated higher invasion compared to the sphere phenotype. HPF is defined as a High Power Field or a 20x Field of Vision. *n* = 6 for all conditions. ^ *p* < 0.05, + *p* < 0.01, *p < 0.001.

We then tested cellular behavior using a Matrigel invasion assay to compare both the migration and invasion of cells of both adherent and sphere phenotypes. Within each patient derived glioblastoma cell culture, cells in the adherent phenotype displayed both higher migration towards FBS and higher invasion through a Matrigel matrix (Figure 2).

### Sphere phenotype shows higher chemoresistance

**Figure 3:**
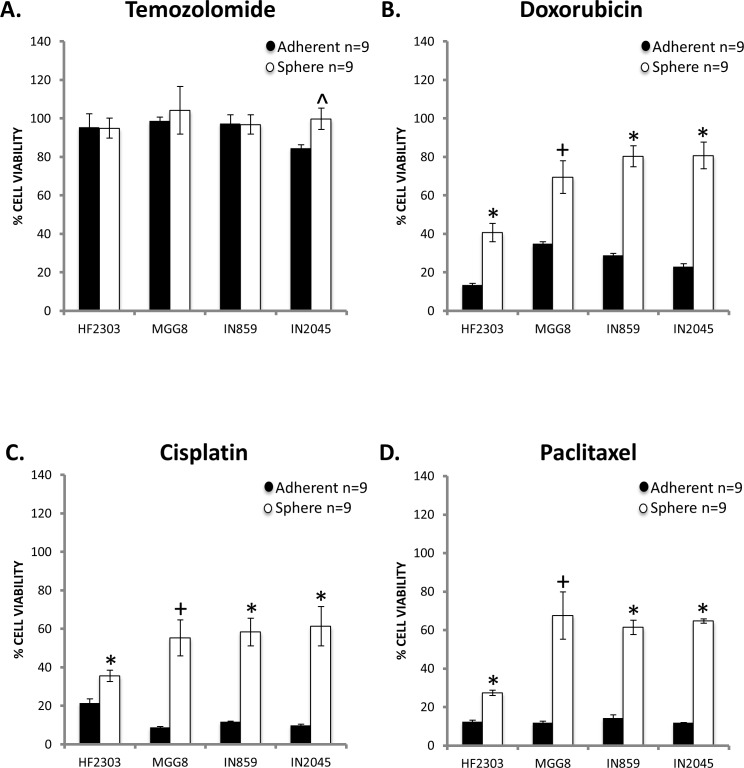
**A.** Temozolomide (25ug/mL) had little effect on either the adherent or sphere phenotype across patient derived glioblastoma cell cultures. Temozolomide was minimally effective against the IN2045 adherent phenotype. Doxorubicin (1ug/mL) **B.**, Cisplatin (40ug/mL) **C.**, and Paclitaxel (120ug/mL) **D.** showed efficacy against both the adherent and sphere phenotype but across patient derived glioblastoma cell cultures and chemotherapeutic agents, the sphere phenotype was more chemoresistant than the adherent phenotype. *n* = 9 for all conditions. ^ *p* < 0.05, + *p* < 0.01, *p < 0.001.

Next, we studied the *in vitro* sensitivity to chemotherapeutic agents temozolomide, cisplatin, paclitaxel, and doxorubicin to compare the chemosensitivity of the adherent and sphere phenotypes. For all four patient derived glioblastoma cell cultures, temozolomide showed no effect in either the adherent or sphere phenotype, with the exception of a small effect in the IN2045 adherent phenotype (Figure 3A). Temozolomide was inactive in all other groups. Doxorubicin was cytotoxic for all patient derived glioblastoma cell cultures tested (Figure 3B), and the sphere phenotype was more resistant to doxorubicin. This difference can be explained by the cell cycle dependency of doxorubicin's cytotoxicity (Figure 1). An effective response was seen for cisplatin and paclitaxel, two DNA damaging agents, for all four patient derived glioblastoma cell cultures and both phenotypes (Figure 3C-3D). HF2303 spheres were highly sensitive to paclitaxel. For all patient derived glioblastoma cell cultures and treatments the sphere phenotype was more resistant to chemotherapy than the adherent phenotype.

### *In vivo* behavior of adherent and sphere phenotypes

**Figure 4:**
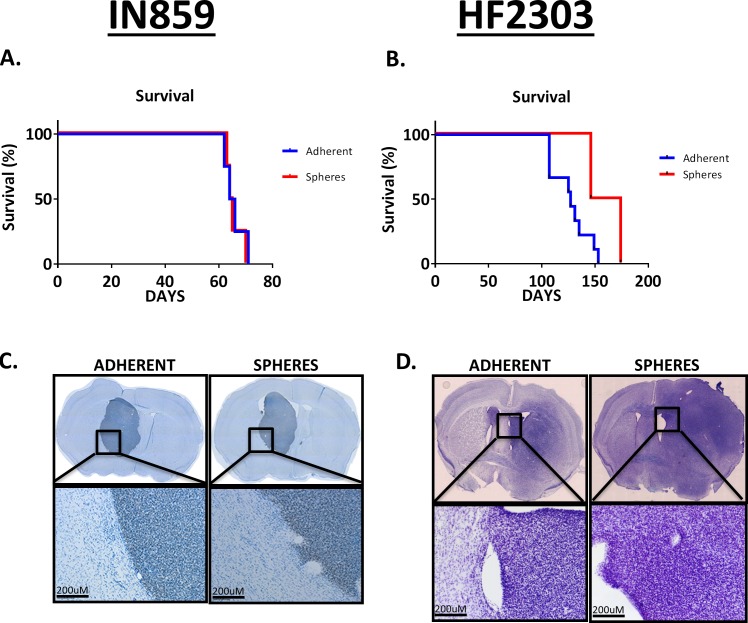
**A-B.** 30,000 sphere phenotype cells or adherent phenotype cells were injected into the striatum of adult RAG1−/− mice. Survival time was roughly double IN859 for HF2303. **C.**-**D.** Tissue sections were stained for human nuclear antigen to identify implanted human glioma cells. Tumor morphology was similar whether the sphere phenotype or adherent phenotype cells were injected. Tumor borders for IN859 tumors were well-demarcated and non-invasive. Scale bars 200 μm. Conversely, tumor borders for HF2303 were poorly demarcated and highly invasive with cells appearing to invade the surrounding parenchyma in a perivascular fashion. *n* = 4 for both adherent and sphere conditions in IN859 and *n* = 7 for the adherent HF2303 group and *n* = 2 for the sphere HF2303 group.

We hypothesized that, given the different *in vitro* behavior of these phenotypes, when implanted into the brain, they might behave distinctly. We tested this hypothesis by implanting glioma cells grown adherently or as spheres into the striatum and studying their *in vivo* growth. For IN859 cells, the survival was similar for both groups (Figure 4A), with mean survival being 65.8 days for the adherent cells and 65.5 days for the sphere cells. For the HF2303 cells, the survival was comparable (Figure 4B) for both groups with mean survival being 135 days for the adherent phenotype and 150 days for the sphere phenotype. In addition to having similar survival times, both IN859 tumor types showed similar morphology, as did the HF2303 tumors. IN859 tumors showed more homogeneous growth with well demarcated, non-invasive borders (Figure 4C). In contrast, HF2303 tumors showed more irregular growth with poorly demarcated, highly invasive borders (Figure 4D).

### *In vitro* reversible transition between adherent and sphere phenotypes

**Figure 5:**
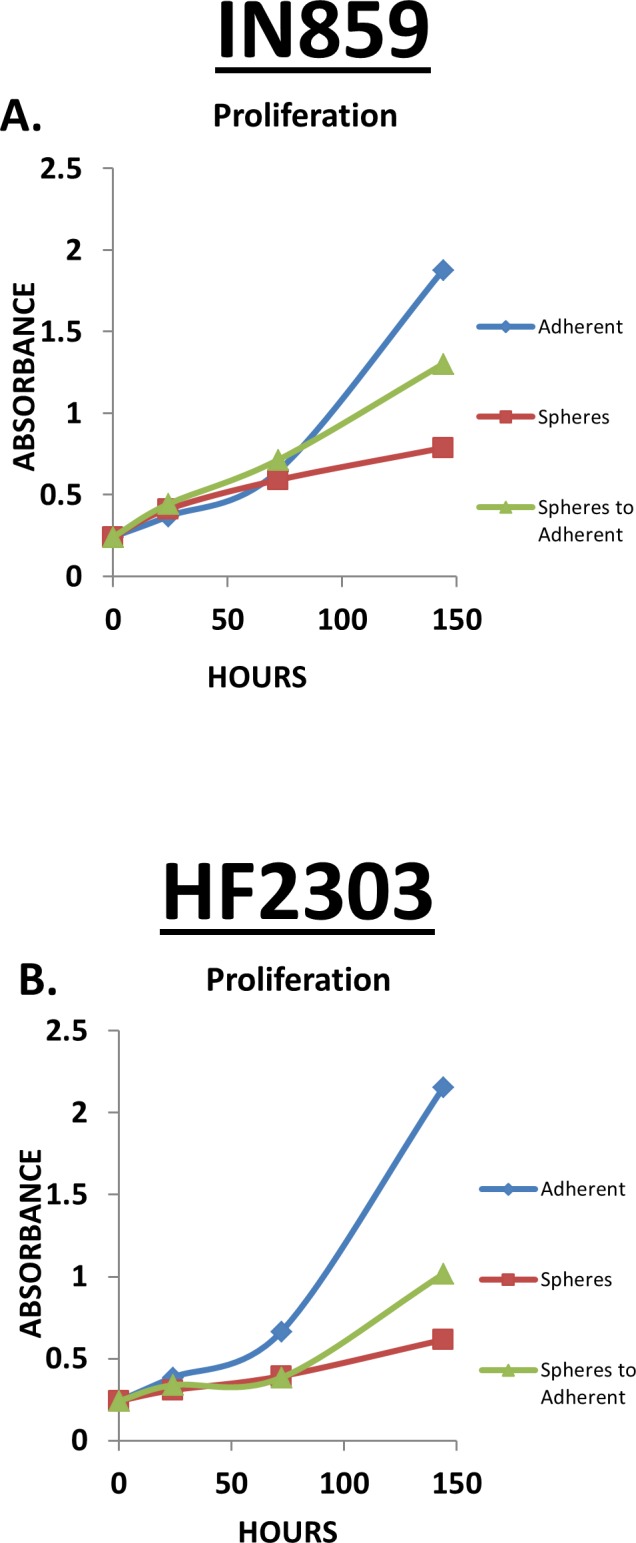
**A-B.** IN859 and HF2303 adherent cells showed a higher rate of proliferation compared to the sphere phenotype. When sphere phenotype cells were placed into adherent conditions, at approximately 72 hours the cells started to proliferate at a rate similar to the adherent phenotype. *n* = 3 for all conditions.

**Figure 6:**
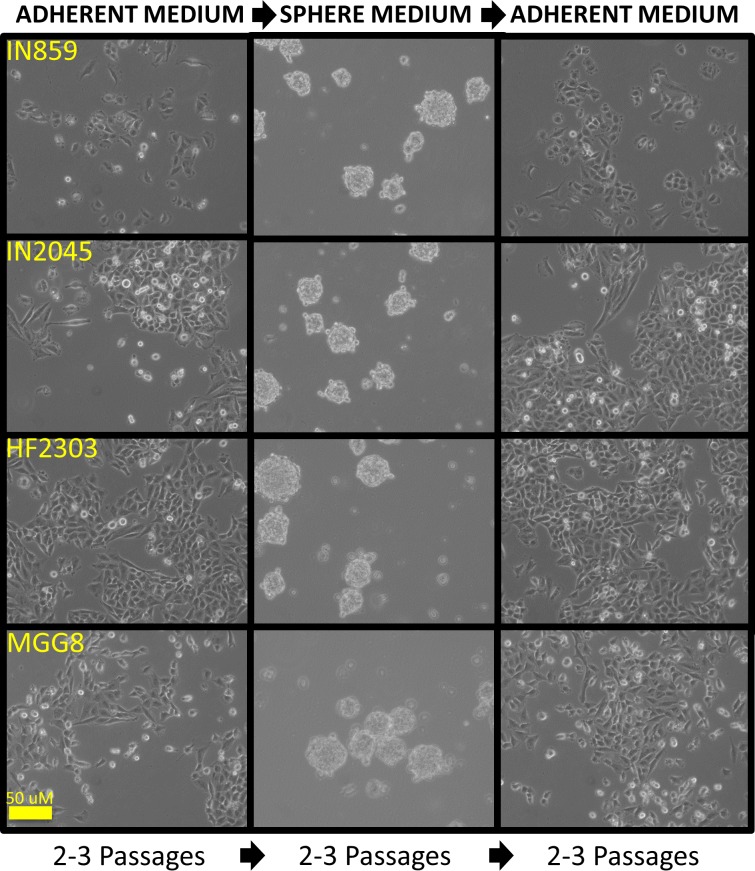
Glioma patient derived glioblastoma cell cultures when cultured in serum containing medium grew as an adherent monolayer When cells were passaged into serum-free medium containing EGF/FGF, cells grew as neurosphere-like tumorspheres. When these cells were passaged back into serum containing medium, the cells again grew as an adherent monolayer. This process could be repeated demonstrating the reversibility of each phenotype.

Both phenotypes of glioma cells displayed similar *in vivo* behavior after being placed in the brain microenvironment and grown as tumors. As *in vitro* they display different behaviors, and *in vivo* they behave similarly, we hypothesized that the phenotypes *(in vitro v. in vivo)* are reversible. We tested this hypothesis by comparing the proliferation rate of sphere cells when placed into adherent medium with baseline growth of adherent and sphere cells in their corresponding medium (Figure 5A-5B). In this experiment sphere cells plated into adherent medium increased their proliferation, illustrating the capacity of sphere cells to become adherent cells (Figure 5). Comparable phenotypic changes were seen for all cells *in vitro*. All patient derived glioblastoma cell cultures were shown to revert to a neurosphere morphology when placed into sphere medium, and into an adherent morphology when placed into the adherent medium (Figure 6).

### qPCR measurments of glioma-specific proteins demonstrate a reversible transition between states

**Figure 7:**
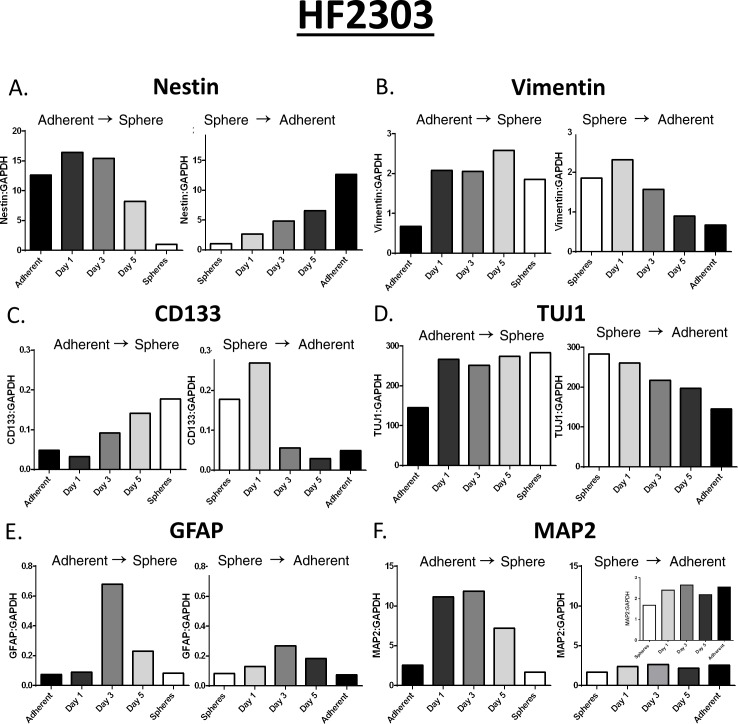
Several of the mRNAs (ie. Nestin, Vimentin, CD133, TUJ1) measured changed depending on the state of the cell (Figure 7A-D) The remainder of the mRNAs (GFAP, MAP2) did not change between phenotypes but seemed to spike in the middle of the transition between phenotypes (Figure 7E-F). ΔΔCt Values were shown for mRNA readings in this figure.

Our data so far suggested that the overall phenotypes (i.e., proliferation, invasion, chemotaxi, morphology, etc.) were reversible (i.e, cells could transition from spheres to adherent, and vice-versa). These data suggested that the expression of individual mRNAs should also differ, and be reversible between phenotypes. To test this we performed qPCR on a selected panel of mRNAs of importance in glioma stem cell function: CD133, nestin, vimentin, TUJ1, GFAP and MAP2. The level of expression of mRNAs for nestin, vimentin, CD133, and TUJ changed between both states. The levels of expression of mRNAs for GFAP and MAP2 did not vary between phenotypic states, but showed a peak in expression during the transition from one state to the other (Figure 7).

### Microarray analysis reveals gene expression landscape of adherent versus sphere phenotype

**Figure 8:**
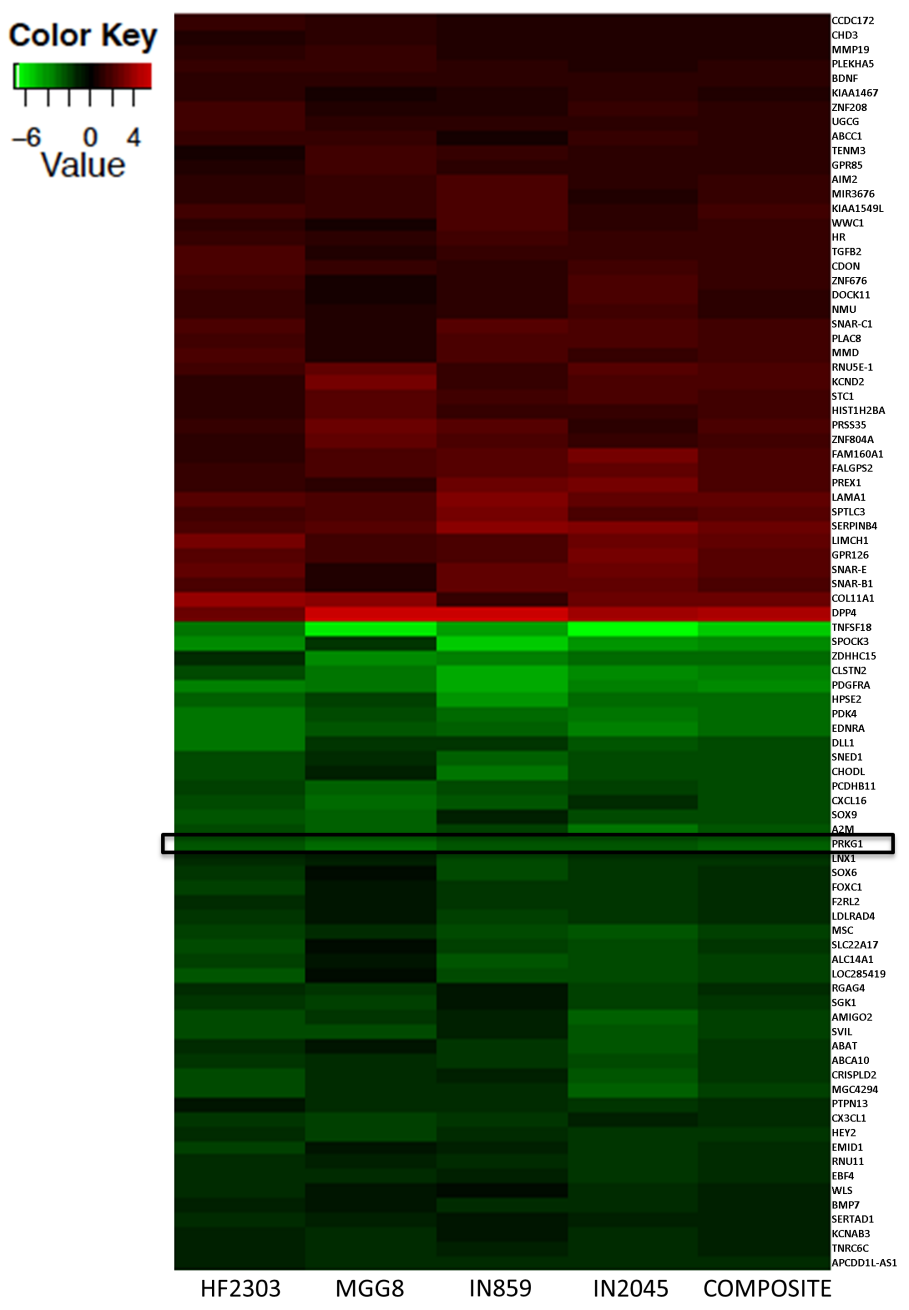
The genetic expression profiles of the sphere phenotype were compared to those of the adherent phenotype for HF2303, MGG8, IN859, and IN2045 Negative values (green) represent genes overexpressed in the spheres relative to the adherent phenotype while positive values (red) represent genes overexpressed in the adherent phenotype relative to the spheres. Values are presented as log_2_ relative change. A list of differentially expressed genes was generated for each patient derived glioblastoma cell culture and across patient derived glioblastoma cell cultures including PRKG1 which was overexpressed in the sphere phenotype across patient derived glioblastoma cell cultures.

We next sought to compare the genetic profiles of each phenotype using cDNA microarray analysis to understand differential gene expression between both phenotypes. Figure 8 shows the top 100 genes that were differentially expressed between the adherent and sphere phenotypes across HF2303, MGG8, IN859, and IN2045 patient derived glioblastoma cell cultures. Note that the location of cGMP-dependent protein kinase 1 (PRKG1) is highlighted. This gene was overexpressed in the four cells studied, and was further examined as shown below.

### Detailed exploration of networks enriched in the sphere phenotype highlights novel potential pharmacological approaches to treat GBM

**Figure 9:**
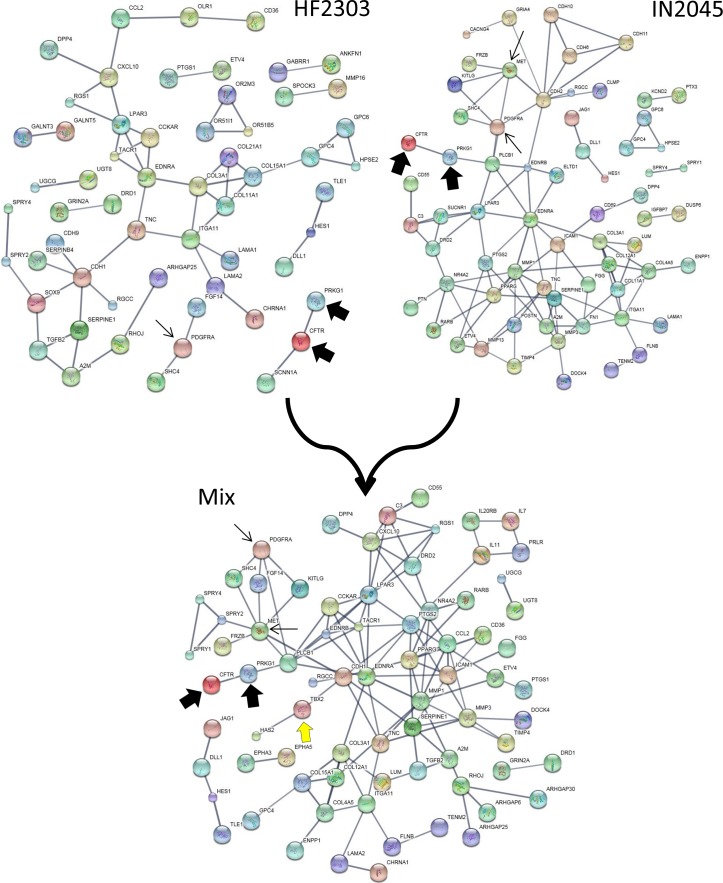
Enrichment networks of tumorspheres (vs. adherent cultures) for HF2303 and IN2045 are shown in the upper half The mixed network is shown in the lower half. Note that CFTR is very strongly associated with PRKG1 suggesting that CFTR could be a viable ion channel to inhibit as a potenital anti-glioma agent. In the mix network note that there are further genes that now have become closely associated with PRKG1, which are known to stimulate glioma growth (i.e., *PDGFRA*, *met*), or could constitute novel molecules to explore therapeutically, i.e., *CFTR*, *PLCB1*, and *TBX2*. Details on the construction of networks is given in Materials and Methods.

We explored the genomic landscape of HF2303 and IN2045 glioma stem cells to discover further potential therapeutic targets. From our microarray expression database we extracted the 100 most highly expressed genes and the 100 most highly inhibited genes when gene expression from the sphere condition was compared to the adherent condition. This set of genes, which thus contains the genes most upregulated and those most downregulated in cells grown as tumorspheres, did contain PRKG1 as expected. This study was conducted for the human glioma cell lines HF2303 and IN2045. The resulting networks are illustrated in Figures 9, 10 and Table 1. The HF2303 network displayed a protein-protein interaction (PPI) enrichment p-value of 0.00162. The IN2045 network displayed a PPI enrichment p-value of 1.62×10^−11^. In both networks PRKG1 was strongly associated with CFTR. We identified the gene ontologies which contained both PRKG1 and CFTR, and used the genes enriched within these three ontologies to construct a combined network (Mix in Figure 9). Data on the effetcs of PRKG1 are shown in Figures 11 and 12. The combined network's PPI enrichment p-value was 0. This network includes mRNAs from genes already known to be of importance in glioma biology, e.g., PDGFRA, met, and TGF-β2. The combined network also indicates novel genes that ought to be explored as future potential targets for the treatment of glioblastoma multiforme. We highlight the cystic fibrosis transmembrane regulator (CFTR), the phospholipase PLCB1, and the transcription factor TBX2, amongst others to be found in Figure 9. Very little information is currently known about the activities of these gene products in glioma biology. Figures 10a, 10b and Table 1 show the gene ontology networks that correspond to the gene Figure 9. The gene ontologies and the gene ontology networks illustrate the close correlation of genes involved in brain and neuronal development and genes activating transcription factor function, all of which are enriched within the sphere phenotype of two human glioma stem cell lines.

**Figure 10a:**
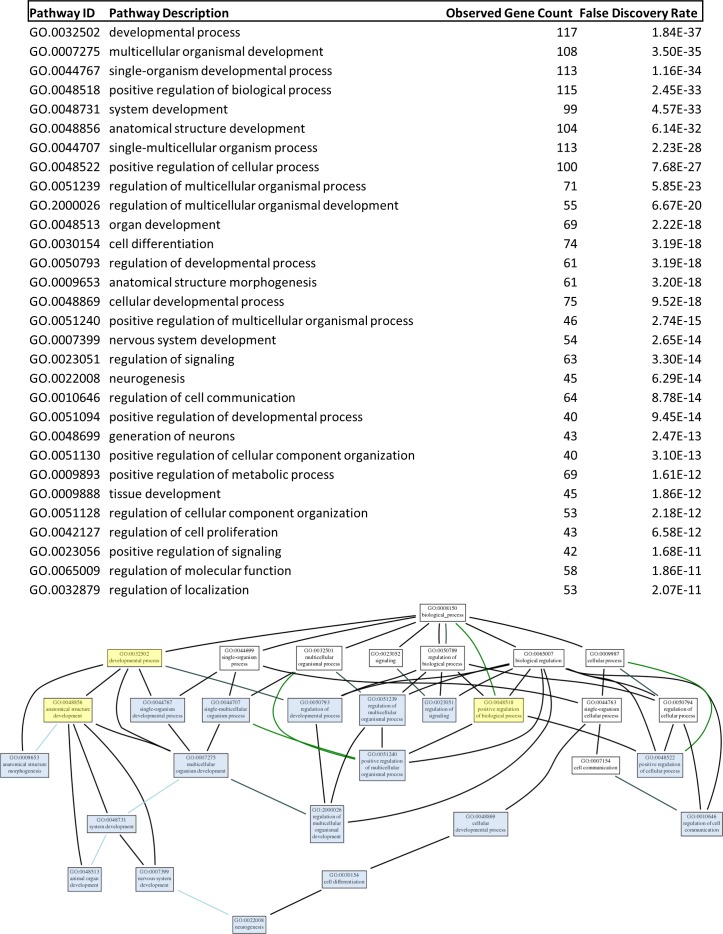
The thirty most statistically significant gene ontologies are shown in the list at the top of the figure and the network organization of these gene ontologies is shown in the bottom half of the figure In constructing this GO network notice how the gene ontology “neurogenesis” is overseeing all other ontologies enriched in the mixed network. This strongly suggests that genes within this ontology and linking to neurogenesis function could be mined for further novel genes for therapeutic targeting for the treatment of glioma.

**Figure 10b:**
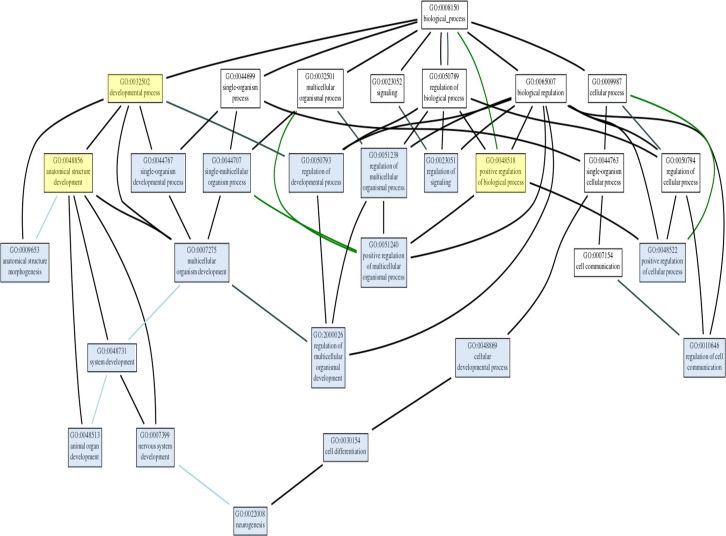
A higher magnification view of the gene ontology network shown at the bottom of Figure 12 is shown here to clarify the evaluation of this network

**Figure 11:**
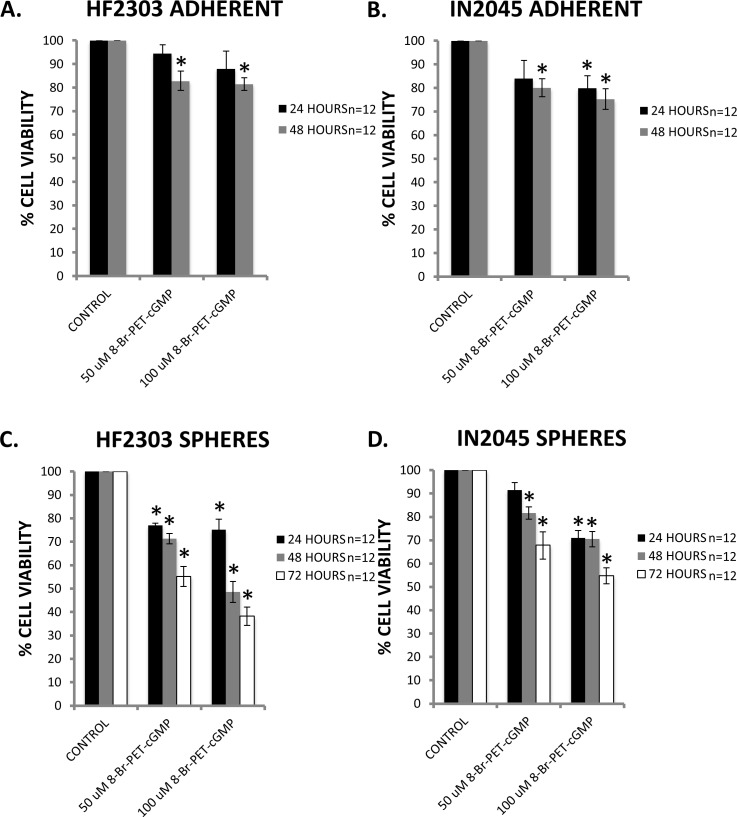
**A-B.** Specific activation of PRKG1 by the cGMP analogue 8-Br-PET-cGMP resulted in a small, dose-dependent reduction in cell viability in HF2303 and IN2045 adherent phenotype cells. **C.**-**D.** Similarly, activation of PRKG1 showed a dose-dependent reduction in cell viability in HF2303 and IN2045 sphere phenotype cells. The reduction in cell viability was more marked compared to the adherent phenotype cells. *n* = 12 for all conditions. ^ *p* < 0.05, + *p* < 0.01, *p < 0.001.

**Figure 12:**
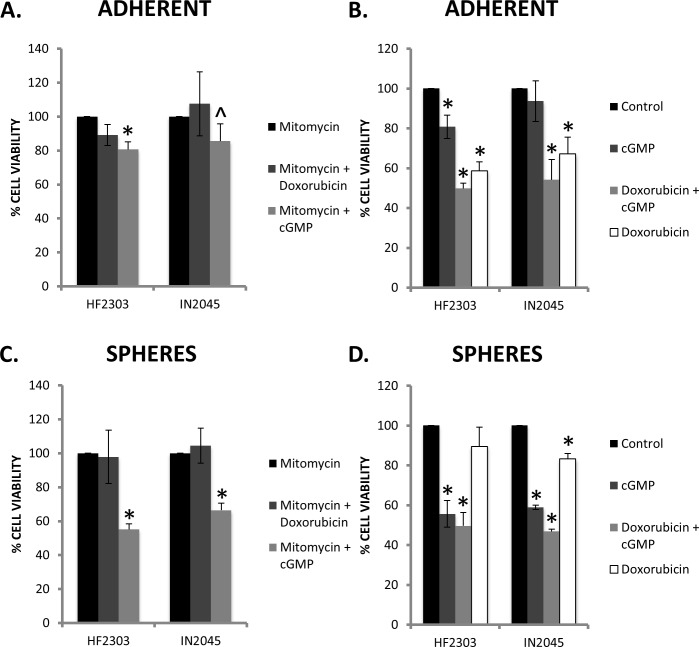
**A.** The addition of mitomycin abrogated the effect of doxorubicin on HF2303 and IN2045, whereas the addition of mitomycin did not modify the response to activation of PRKG1 by the cGMP analogue 8-Br-PET-cGMP. **B.** In HF2303 and IN2045 adherent cells, doxorubicin showed a more dramatic decrease in cell viability compared to activation of PRKG1. However the addition of both doxorubicin and 8-Br-PET-cGMP showed a further reduction in cell viability than either alone. **C.** Similar to the adherent phenotype, the addition of mitomycin abrogated the effect of doxorubicin on HF2303 and IN2045 spheres, whereas the addition of mitomycin did not modify the response to activation of PRKG1 by 8-Br-PET-cGMP. The effect of PRKG1 was markedly greater in the sphere phenotype compared to the adherent phenotype. **D.** Converse to the adherent phenotype, in the sphere phenotype, activation of PRKG1 showed a more marked reduction in cell viability compared to treatment with doxorubicin. The addition of doxorubicin and 8-Br-PET-cGMP showed a more dramatic reduction in cell viability than either agent alone. *n* = 3 for all conditions. ^ *p* < 0.05, + *p* < 0.01, *p < 0.001.

**Table 1:** 

HF2303				HF2045			
Pathway ID	Pathway Description	Observed Gene Count	False Discovery Rate	Pathway ID	Pathway Description	Observed Gene Count	False Discovery Rate
GO.0030198	extracellular matrix organization	17	1.52E-05	GO.2000026	regulation of multicellular organismal development	46	2.94E-10
GO.0042127	regulation of cell proliferation	33	2.55E-05	GO.0051239	regulation of multicellular organismal process	55	5.29E-09
GO.0007267	cell-cell signaling	25	6.39E-05	GO.0048731	system development	69	8.03E-09
GO.0044700	single organism signaling	66	8.11E-05	GO.0007275	multicellular organismal development	73	4.30E-08
GO.0030155	regulation of cell adhesion	19	0.000115	GO.0051094	positive regulation of developmental process	35	4.30E-08
GO.0007154	cell communication	66	0.000146	GO.0050793	regulation of developmental process	48	1.52E-07
GO.0040011	locomotion	27	0.000218	GO.0009888	tissue development	40	2.72E-07
GO.0048731	system development	52	0.000218	GO.0032502	developmental process	77	2.72E-07
GO.0050678	regulation of epithelial cell proliferation	13	0.000218	GO.0044767	single-organism developmental process	76	3.94E-07
GO.0048514	blood vessel morphogenesis	15	0.000243	GO.0030198	extracellular matrix organization	19	5.34E-07
GO.0048518	positive regulation of biological process	64	0.000358	GO.0040011	locomotion	34	5.34E-07
GO.0007275	multicellular organismal development	56	0.000375	GO.0051240	positive regulation of multicellular organismal process	36	6.66E-07
GO.0008284	positive regulation of cell proliferation	21	0.000447	GO.0044707	single-multicellular organism process	83	6.92E-07
GO.0001944	vasculature development	16	0.000492	GO.0048856	anatomical structure development	69	1.15E-06
GO.0007155	cell adhesion	23	0.000492	GO.0048699	generation of neurons	35	1.52E-06
GO.0009653	anatomical structure morphogenesis	36	0.000492	GO.0051960	regulation of nervous system development	24	3.61E-06
GO.0044707	single-multicellular organism process	67	0.000492	GO.0022603	regulation of anatomical structure morphogenesis	27	4.08E-06
GO.0065009	regulation of molecular function	42	0.000506	GO.0022008	neurogenesis	35	4.64E-06
GO.0040012	regulation of locomotion	19	0.000648	GO.0009653	anatomical structure morphogenesis	44	5.25E-06
GO.0051239	regulation of multicellular organismal process	38	0.000875	GO.0051130	positive regulation of cellular component organization	31	6.74E-06
GO.0001568	blood vessel development	15	0.000956	GO.0048468	cell development	37	1.17E-05
GO.0072358	cardiovascular system development	20	0.000956	GO.0030154	cell differentiation	55	1.28E-05
GO.0072359	circulatory system development	20	0.000956	GO.0048513	organ development	50	1.28E-05
GO.0044708	single-organism behavior	14	0.000961	GO.0030155	regulation of cell adhesion	21	1.46E-05
GO.0023051	regulation of signaling	41	0.00118	GO.0048522	positive regulation of cellular process	68	1.50E-05
GO.1903053	regulation of extracellular matrix organization	5	0.00122	GO.0040012	regulation of locomotion	23	1.69E-05
GO.0030334	regulation of cell migration	17	0.00125	GO.0048518	positive regulation of biological process	74	2.31E-05
GO.0016477	cell migration	19	0.00135	GO.0070482	response to oxygen levels	15	2.65E-05
GO.0050680	negative regulation of epithelial cell proliferation	8	0.00135	GO.0030334	regulation of cell migration	21	2.90E-05
GO.0010646	regulation of cell communication	42	0.00142	GO.0006928	movement of cell or subcellular component	32	2.99E-05
GO.0040017	positive regulation of locomotion	13	0.00163	GO.0016477	cell migration	23	5.48E-05
GO.0007399	nervous system development	33	0.00185	GO.0051962	positive regulation of nervous system development	17	5.89E-05
GO.0032502	developmental process	58	0.00185	GO.0048870	cell motility	24	6.04E-05
GO.0032879	regulation of localization	36	0.00185	GO.0051674	localization of cell	24	6.04E-05
GO.2000026	regulation of multicellular organismal development	28	0.00199	GO.0007155	cell adhesion	26	7.39E-05
GO.0007423	sensory organ development	15	0.00204	GO.0070372	regulation of ERK1 and ERK2 cascade	12	7.67E-05
GO.0050790	regulation of catalytic activity	35	0.00213	GO.0032879	regulation of localization	43	8.70E-05
GO.0009887	organ morphogenesis	20	0.0023	GO.0051270	regulation of cellular component movement	22	8.70E-05
GO.0007610	behavior	15	0.00244	GO.0044700	single organism signaling	71	0.000112
GO.0003008	system process	29	0.00309	GO.0045597	positive regulation of cell differentiation	23	0.00013
GO.0006928	movement of cell or subcellular component	25	0.00309	GO.0050803	regulation of synapse structure or activity	12	0.000133
GO.0022008	neurogenesis	26	0.00309	GO.0007506	gonadal mesoderm development	4	0.000138
GO.0048513	organ development	39	0.00309	GO.0060485	mesenchyme development	11	0.000143
GO.0051674	localization of cell	19	0.00309	GO.0060284	regulation of cell development	22	0.000174
GO.0030335	positive regulation of cell migration	12	0.00316	GO.0098742	cell-cell adhesion via plasma-membrane adhesion molecules	11	0.000174
GO.0048522	positive regulation of cellular process	54	0.00337	GO.0007156	homophilic cell adhesion via plasma membrane adhesion molecules	10	0.000192
GO.0048856	anatomical structure development	52	0.00345	GO.0035295	tube development	19	0.000196
GO.0050806	positive regulation of synaptic transmission	7	0.00345	GO.0001666	response to hypoxia	13	0.000213
GO.0016525	negative regulation of angiogenesis	6	0.00358	GO.0022617	extracellular matrix disassembly	9	0.000245

### PRKG1 stimulation reduces cell viability

PRKG1 is differentially overexpressed in the sphere phenotype in four human glioma stem cell lines, i.e., HF2303, MGG8, IN859 and IN2045. In all cell lines, PRKG1 was within the 100 genes with the highest differential expression between the sphere and the adherent phenotype. If we assume that PRKG1 overexpression is a result of reduced activation, we reasoned that activation of PRKG1 could induce its pro-apoptotic effects. PRKG1 can be permanently activated using the cGMP analogue 8-Br-PET-cGMP. We tested whether activation of PRKG1 would result in decreased cell viability in our model. For the adherent phenotype, activation of PRKG1 reduced cell viability compared to control in both patient derived glioblastoma cell cultures (Figure 11A-11B). The effect was greater for the sphere phenotype, and activation of PRKG1 resulted in reduced cell viability in both patient derived glioblastoma cell cultures (Figure 11C-11D). Maximal effect was observed in HF2303 spheres at 72 hours with a dose of 100 μM 8-Br-PET-cGMP which resulted in an over 50% reduction in cell viability compared to control.

### PRKG1 effects occur in a cell-cycle independent fashion

Mitomycin was used to arrest the cell cycle and cell proliferation to test whether the effect of PRKG1 was cell cycle dependent or independent. For both HF2303 and IN2045, the effect of doxorubicin (an inhibitor of topoisomerase II, and cell-cycle dependent cytotoxin) was abrogated by mitomycin. Conversely, mitomycin had no effect on treatment with 8-Br-PET-cGMP suggesting that downstream reduction in cell viability seen with PRKG1 activation occurs in a cell cycle independent manner (Figure 12A, 12C).

As we have shown, the sphere phenotype is normally more resistant to traditional agents such as doxorubicin (Figure 12D), whereas the adherent phenotype shows a much higher sensitivity to doxorubicin (Figure 12B). The converse is true for PRKG1 activation, where the sphere phenotype is more sensitive (Figure 12B, 12D). When PRKG1 activation is added to doxorubicin, in both the adherent and sphere phenotype there is a reduction in cell viability compared to either PRKG1 activation or doxorubicin alone. This suggests that doxorubicin and 8-Br-PET-cGMP may be inhibiting growth of different cell populations. (Figure 12B, 12D)

## DISCUSSION

The growth of human gliomas is thought to follow the cancer stem cell model. Variable experimental definitions of glioma stem cells *in vitro* has led to discrepancies in the literature concerning the behavior and characterization of glioma stem cells. This is particularly evident in the varying methods used to study and culture glioma stem cells. The most commonly employed methods for culture utilize serum-containing media to generate adherent monolayers, serum-free media containing EGF and bFGF to generate neurosphere-like tumorspheres, and putative increased expression of CD133 in stem cells. Formation of neuro/tumorspheres *in vitro* coupled to *in vivo* tumor growth are the most reliable methods to identify glioma stem cells. Cells growing as spheres are thought to be able to grow as adherent cells if the medium is changed; adherent cells however, are not supposed to be able to grow as spheres.

Unexpectedly our experiments showed that glioma stem cells can grow reversibly (i.e., interchange from one phenotype to the other and vice-versa), but that each phenotype differs in its behavioral characteristics vis-à-vis proliferation, invasion, and chemoresistance.

Our results show that all patient derived glioblastoma cell cultures utilized in this study are capable of growth as an adherent monolayer and as tumorspheres. These growth patterns were reversible by transferring cells from adherent conditions to sphere conditions or vice versa. For all glioma stem cells, cells cultured under adherent conditions displayed a higher rate of proliferation, higher rate of invasion, and lower chemoresistance compared to the same cells cultured under sphere conditions. The exception was the chemosensitivity assays performed with temozolomide which showed little cytotoxicity in either the adherent or sphere phenotypes. These observations imply that these assays are dependent on the culture method employed. Thus, these results serve to point out that interpretation of *in vitro* studies involving glioma stem cells is highly dependent on the method of study being employed.

Reversibility of sphere and adherent phenotypes led us to predict that if cells were placed in similar microenvironments *in vitro* or *in vivo* their behavior may equally become alike. *In vivo* we were able to test this hypothesis by implanting equal numbers of cells into the striatum of mice. Tumor growth rate was similar for both groups. This suggests that the sphere-state and adherent-state, at least for the cells tested, are reversible *in vivo*. It is thus likely that tumors are composed of cells in various phenotypic states, two of which are the *in vitro* sphere and adherent phenotypes which are reversible.

Based on these results, we propose a simple experimental definition of glioma propagating/initiating cells (GPCs). We propose that all cells referred to as GPCs should grow reversibly as an adherent monolayer and as tumor spheres *in vitro* and should form tumors following *in vivo* implantation. Utilizing this simple experimental definition may facilitate inter-laboratory experimental replication. Also, work from other investigators can easily prove, disprove, expand, or amend this definition. We also suggest that assays be interpreted in the light of the phenotype used. Utilizing this definition we were able to identify a new therapeutic target to be used with standard treatment of care such as chemotherapy and radiation.

Genetic expression profiles were used to evaluate the genes/signaling pathways underlying the state-specific behaviors, and to compute comparative gene expression maps to identify actionable targets for specific killing of cells in the chemoresistant sphere-state. cDNA microarrays were used to determine the genetic expression profile of the sphere and adherent phenotypes and to generate a list of genes over- and underexpressed in each phenotype. Expression of various mRNAs (i.e., CD133 and others) was also reversibly expressed between the adherent and sphere phenotype.

One gene that was selectively overexpressed in the sphere phenotype was cGMP dependent protein kinase 1 (PRKG1). Review of the literature revealed previous reports of involvement of PRKG1 in apoptosis of cancer cells via hyperactivation of death-associated protein kinase 2 [20–22]. We hypothesized that overexpression of PRKG1 in the sphere phenotype could be a compensatory mechanism for low endogenous activation. We thus assumed that if increased amounts of PRKG1 could be exogenously activated this might induce apoptosis in the normally resistant PRKG1 overexpressing spheres. Our experimental results support our assumptions.

Analysis of the networks formed by HF2303 or IN2045 demonstrated a close association of PRKG1 with CFTR, two proteins not yet linked to glioblastoma biology. Ontologies containing both genes were used to create a combined network. This combined network linked both PRKG1 and CFTR to genes already known to be of importance in glioma biology such as PDGFRA, met, and TGFβ2, amongst others. This provides support for the soundness and biological significance of the protein-protein interaction networks (PPI) and gene ontology networks uncovered. Further, this implies that mining of these networks will identify novel potential therapeutic molecules. Examination of the PPI interaction networks suggests that the ion channel CFTR, the phospholipase PLCB1, and the transcription factor TBX2 ought to be considered novel potential proteins to be inhibited for the treatment of glioblastoma. The ontology networks providing the functional integration of network genes affords further support for the validity of this analysis by highlighting ontologies related to neuronal development, and activating transcription factor activity.

8-Br-PET-cGMP is a highly specific activator of both the A and B isoforms of cGMP-dependent protein kinase I (with very poor activation of type II). Treatment of cells with 8-Br-PET-cGMP, i.e., activation of PRKG1, resulted in a significant and dose-dependent reduction of cell viability in the sphere phenotype that overexpresses PRKG1. This suggests that the observed reduction in cell viability is due to activation of apoptosis.

Most traditional chemotherapeutic agents are cell cycle dependent as they damage DNA or disrupt components of the cell division machinery resulting in cell death. Doxorubicin, for example, stabilizes the topoisomerase II complex preventing cell replication. When pretreated with mitomycin to shut down the cell cycle, the killing effect of doxorubicin was abrogated. However, when cells were pretreated with mitomycin the cytocidal effect of activation of PRKG1 was not abrogated, especially within spheres. This suggests that, in comparison to most traditional chemotherapeutic agents, the effect of activation of PRKG1 ocurs in a cell cycle independent fashion. In addition, the combination of PRKG1 activation and doxorubicin led to a larger reduction in cell viability than either alone. Together with the observation that doxorubicin works in a cell cycle dependent fashion, while activation of PRKG1 is likely to work in a cell cycle independent fashion, suggests that activation of PRKG1 may be acting on cells in a different phenotype and state, i.e., slowly dividing sphere glioma cells, than doxorubicin.

PRKG1 appears to be a novel therapeutic phenotype-specific drug for the treatment of glioblastoma. It warrants future *in vivo* testing to determine whether activation of PRKG1 reduce tumor burden and extends survival either alone or in combination with standard therapy. Its ability to kill the normally chemoresistant sphere phenotype while traditional chemotherapy agents are more efficacious for the adherent phenotype combined with observation that activation of PRKG1 may be inhibiting a different subpopulation of cells than traditional chemotherapeutic agents make it a particularly attractive addition for testing in combination with traditional therapies. Moreover, we have potentially identified a new method for generating candidate therapeutic agents and have generated a list of up- and downregulated genes in each phenotype that can be further tested as therapeutic candidates.

In summary, we illustrate the reversibility of human glioma stem cells *in vitro* between a sphere phenotype and an adherent phenotype, and that this morphological phenotype correlates with cellular proliferation rate, invasion capability, and response to chemotherapy. However, in a comparable microenvironment (e.g., normal brain) both phenotypes grew tumors at comparable rates; this result is predicted by the phenotype reversibility detected. Finally, the differential genomic landscape between phenotypes was utilized to identify a novel potential drug that inhibits selectively the sphere phenotype for which few pharmacological agents exist. PPI and gene ontology networks examined suggest the existence of further targets to be explored for the treatment of glioblastoma, such as PRKG1, CFTR, PLCB1, and TBX2.

## MATERIALS AND METHODS

### Patient derived glioblastoma cell cultures

The HF2303 human gliosarcoma patient derived glioblastoma cell culture was obtained from Dr. Tom Mikkelsen at Henry Ford Hospital (Detroit, MI). The MGG8 human glioma patient derived glioblastoma cell culture was obtained from Samuel Rabkin, Harvard University. The IN859 and IN2045 patient derived glioblastoma cell cultures were obtained from Dr. John Darling, University College London. Experiments described in detail below were all performed in triplicate.

### Animal strains

Six to eight week-old male/female Rag1^tm1Mom^/J were purchased from Jackson Laboratory (Bar Harbor, ME) and subsequently bred in-house to maintain a colony. All animal procedures were approved by the University Committee on Use and Care of Animals (UCUCA) at the University of Michigan.

### Cell proliferation assays

CellTiter 96^®^ Aqueous Non-Radioactive Cell Proliferation Assay reagents were purchased from Promega (Madison, WI). For each patient derived glioblastoma cell culture, 7500 cells were plated in 100 μL of media in each well of a 96-well plate. Cells were plated in four wells per experimental group. Additionally, blank wells were plated consisting of medium alone. At 24, 48, and 72 hours MTS solution was added to each well and incubated for 3 hours at 37°C in a 5% CO_2_ humidified atmosphere. After colorimetric development, the plate was read at 490 nm on a PerkinElmer EnSpire 2300 Multimode Plate Reader (Waltham, MA). The average blank value was then subtracted from each experimental well.

### Matrigel invasion assay

BD BioCoat^TM^ BD Matrigel^TM^ Invasion Chamber inserts with 8 μm pores and BD BioCoat^TM^ Control inserts with 8 μm pores were purchased from BD (Franklin Lakes, NJ). Inserts were rehydrated in medium for 2 hours at 37°C in a 5% CO_2_ humidified atmosphere. The medium was then removed. In the bottom of a 24 well plate 750 μL of DMEM supplemented with 10% FBS was placed as the chemoattractant. The inserts were then placed into each well of the 24 well plate. On the interior of the inserts, 25,000 cells in 500 μL of serum-free DMEM were plated. Plates were then incubated for 24 hours at 37°C in a 5% CO_2_ humidified atmosphere. After incubation, the non-invading cells were removed from the upper surface of the membrane by scrubbing the membrane with a cotton-tipped swab. The membranes were then cut out of the inserts and fixed and stained with Differential Quik Stain (Polysciences, Inc.; Warrington, PA). The number of invading/migrating cells was then quantified by counting the number of cells per 20X field. Five random fields were counted per well and three wells were used for each experimental group. The experiment was repeated twice. Migrating cells were those cells present on the under surface of the control inserts. Invading cells were those cells present on the under surface of the Matrigel inserts. An invasion index was calculated by taking the mean number of invading cells, dividing by the mean number of migrating cells, and multiplying by 100.

### Chemosensitivity assays

Temozolomide, paclitaxel, cisplatin, and doxorubicin were purchased from Sigma-Aldrich (St. Louis, MO). A total of 5000 cells in 100 μL were plated into each well of a 96-well plate. Cells were incubated for 24 hours at 37°C in a 5% CO_2_ humidified atmosphere. After 24 hours, the chemotherapeutic agent was added to each well at the specified concentration. The chemotherapeutic agent was dissolved in media at 11X final concentration and then 10 μL was added to each well. The plates were then incubated for an additional 72 hours. At that time, MTS assays were performed as previously described. Each experimental group was performed in triplicate and the experiment was performed three times. Percent cell viability was calculated by dividing the mean absorbance of the experimental group by the mean absorbance of the control group (no chemotherapeutic agent added) and multiplying by 100.

### Stereotactic tumor implantation and tissue preparation

Six to eight week old female Rag1^tm1Mom^/J mice were utilized for tumor implantation. Mice were anesthetized with intraperitoneal injection of 0.5 mg/kg dexmedetomidine and 75 mg/kg ketamine. Mice were then placed into a stereotactic frame a single midline cranial incision was made. Coordinates based off of bregma were used for placement of a burr hole at +0.5 mm anterior-posterior and +2.5 mm medial-lateral (right). A burr hole was placed at this location exposing the underlying dura which was opened and reflected using a 27 gauge needle. A total of 30,000 cells in 1 μL of DMEM were then injected at a depth of 3.0 mm from the brain surface using a 5 μL Hamilton syringe equipped with a 33-gauge needle. Cells were allowed to settle for 6 minutes prior to slow withdrawal of the needle. The skin was then sutured and reversal agent consisting of atipamezole 1 mg/kg intraperitoneal was given. Post-operative pain relief was achieved via buprenorphine 0.1 mg/kg given subcutaneously. At specified time points or when moribund mice underwent transcardial perfusion oxygenated, heparinized (100 units/L) Tyrode's solution followed by 4% paraformaldehyde (PFA). Brains were harvested and then post-fixed in 4% PFA for 2-3 days. Brains were sectioned using a vibratome yielding 50 μm sections for further analysis.

### Immunohistochemistry

For both DAB peroxidase immunolabeling and immunofluorescence, brain sections were transferred into 12 well plates containing TBS-0.5% Triton X (Tx) for 1 hour while shaking. Antigen retrieval was performed using 10 mM sodium citrate solution 60-90°C for 20 minutes followed by room temperature sodium citrate solution for 20 minutes. For DAB peroxidase staining, sections were then incubated with 5% hydrogen peroxide for 30 minutes to quench endogenous peroxidase activity. After washing with TBS-Tx, non-specific antibody binding was blocked using TBS-Tx with 10% horse serum for 2 hours at room temperature while shaking. Next sections were incubated with primary antibody diluted in TBS-Tx/1% horse serum/0.1% sodium azide at 4°C for 48 hours while continuously shaking. After washing with TBS-Tx, sections were incubated in appropriate biotinylated secondary antibody diluted in TBS-Tx/1% horse serum for 24 hours at 4°C while continuously shaking. Sections were then washed in TBS-Tx and incubated in Vectastain Elite ABC system (Vector Laboratories; Burlingame, CA) AB solution for 6 hours at room temperature. After washing with PBS followed by 0.1 M sodium acetate (pH 6.0), sections were stained in DAB staining solution. Reactions were stopped using 10% azide and then washed sequentially in sodium acetate followed by PBS. Sections were then dehydrated and mounted using Histomount. Anti-human nucleus (1:500, Millipore MAB1281) was used and then developed using secondary biotinylated rabbit anti-mouse (1:1000, Dako E0464).

### Cell culture conditions

All patient derived glioblastoma cell cultures were maintained *in vitro* in 95% air/5% CO_2_ at 37°C under humidified conditions. Culture medium for adherent growth of HF2303, MGG8, IN859, IN2045 consisted of Dulbecco's Modified Eagle Medium (DMEM) supplemented with 10% fetal bovine serum (FBS), 0.3 mg/mL L-glutamine, 50 units/mL penicillin, 50 μg/mL streptomycin, and non-essential amino acids. This medium is referred to as “adherent medium” throughout the manuscript. Cells were passaged every 3-4 days. Culture medium for HF2303, IN859, and IN2045 tumorspheres consisted of DMEM/F12 supplemented with N2 supplement, 50 units/mL penicillin, 50 μg/mL streptomycin, antibiotic/antimycotic (0.5X final concentration), 20 ng/mL epidermal growth factor (EGF), and 20 ng/mL basic fibroblast growth factor (bFGF). Culture medium for MGG8 tumorspheres consisted of Neurobasal Medium supplemented with B27 supplement, 0.45 mg/mL L-glutamine, N2 supplement (0.5X final concentration), 50 units/mL penicillin, 50 μg/mL streptomycin, 20 ng/mL epidermal growth factor (EGF), and 20 ng/mL basic fibroblast growth factor (bFGF). Medium for tumorsphere growth is referred to as “sphere medium” throughout the manuscript. Half culture medium was replaced every 3 days with new EGF and bFGF supplementation. Cells were dissociated using HyClone HyQtase every 6-7 days and split 1:4.

### Rt-qPCR

For real-time quantitative reverse transcription-PCR (RT-PCR) analysis, total RNA was isolated from cells using a NucleoSpin RNA II kit (Macherey-Nagel; Bethlehem, PA). Five micrograms of total RNA were used for reverse transcription. First-strand cDNA was generated using oligo(dT)18 (Fermentas) and Superscript II RT (Invitrogen). Two microliters of the resulting cDNA (1:10 dilution) were used in the real-time reactions with gene-specific primers. The following gene-specific primers were used: Nestin 5′ - 3′ and 5′ - 3′, Vimentin, CD133, TUJ1, GFAP, MAP2, and GAPDH. Quantitative RT-PCR reactions were carried out with FastStart SYBR Green Master mix (Roche) and MACHINE. Fluorescence intensity was measured at the end of each elongation step as a means to evaluate the amount of formed PCR product. GAPDH was used as a reference to normalize the samples.

### cDNA microarray analysis

Total RNA was isolated from HF2303, MGG8, IN859, and IN2045 grown under adherent and sphere conditions using a NucleoSpin^®^ RNA II kit (Macherey-Nagel; Bethlehem, PA). Genetic expression of the cells grown under adherent conditions was compared to expression of cells grown under sphere conditions using an Affymetrix GeneChip® Human Gene 2.0 ST Array. The quantity of a given transcript was compared using a signal log algorithm in two arrays, a baseline array versus a control array. The baseline array in this case was the cells grown under adherent conditions and the experimental array was the cells grown under sphere conditions. The change in the transcript level was then expressed as a log_2_ ratio where a value of 1.0 indicates a 2-fold increase expression of a given gene under adherent conditions and a value of -1.0 represents a 2-fold increase expression of a given gene under sphere conditions. A composite analysis was then performed treating the adherent conditions for all four patient derived glioblastoma cell cultures as the baseline group and the sphere conditions for all four patient derived glioblastoma cell cultures as the experimental group.

### PRKG1 stimulation proliferation assay

Adherent cells were plated in 96 well plates at a density of 1000 cells in 100 μL of medium. Dissociated spheres were plated in 96 well plates at a density of 10,000 cells in 100 μL of medium. Cells were then incubated for 24 hours at 37°C in a 5% CO_2_ humidified atmosphere. At that time, cells were treated daily with 8-Br-PET-cGMP (Axxora P007; Farmingdale, NY) at the specified concentrations in order to activate cGMP-dependent protein kinase 1. After 48 hours from the initial treatment, MTS assays were performed as previously described. Percent cell viability was calculated by dividing the mean absorbance of the experimental group by the mean absorbance of the control group (no treatment) and multiplying by 100. Cells were plated in four wells per experimental group and the experiment was repeated three times.

### Mitomycin proliferation assay

Non-toxic doses of mitomycin that led to cessation of cell proliferation were found for both HF2303 and IN2045 cells. Cells were plated at a density of 100,000 cells/mL in 96 well plates. Cells were allowed 24 hours to equilibrate. At that point, an MTS assay was performed to get a baseline absorbance. To the experimental plate, varying concentrations of mitomycin were added. Cells were allowed to incubate for 48 hours and then an MTS assay was performed. For both HF2303 and IN2045 the non-toxic dose that resulted in no change in absorbance over 48 hours was 10ug/mL (data not shown)

Next, for both HF2303 and IN2045 adherent and sphere a total of 5000 cells in 100ul were plated into each well of a 96 well plate. Cells were incubated for 24 hours at 37*C in a 5% CO2 humidified atmosphere. After 24 hours, mitomycin was added to each well. For those wells receiving doxorubicin or 8-Br-PET-cGMP, cells were preincubated for 6 hours with mitomycin. The doxorubicin concentration used was 0.1ug/mL while the 8-Br-PET-cGMP concentration was 100uM. Cells were then allowed to incubate for 48 hours and an MTS assay was performed.

### Network and gene ontology analysis

The set of 200 genes most upregulated (100) or downregulated (100) in the sphere phenotype v. adherent phenotype in human glioma stem cell lines HF2303 or IN2045 was uploaded into STRING (http://string-db.org/) and networks were evaluated. The minimum required interaction score was set to *high confidence (0.700)*, and the active interaction sources used were: textmining, experiments, databases, neighbourhood, gene fusion, co-occurrence and co-expression. For the view used to prepare the data for Figure 9 disconnected nodes in the network were hidden from view. Figures of the networks, and quantitative network characteristics and gene ontologies were downloaded. The networks are shown in Figure 9, the gene ontologies hierarchy of the combined network are shown in Figures 10a and 10b, and the gene ontologies for the HF2303 and IN2045 networks are illustrated in Table 1. Gene ontologies (GO) were analyzed and visualized using http://geneontology.org and its analysis and visualization tool Amigo2 (http://amigo.geneontology.org/visualize?mode=client_amigo).

### Statistical analysis

Statistical analysis was performed using commercially available software (SPSS version 21, IBM Corp.). For univariate analysis of continuous variables with a normal distribution we planned to utilize 2-sample t-tests, and for continuous variables not meeting the normality assumption we planned to utilize the Mann-Whitney U-test. Data were screened for normality using normality plots and the Shapiro-Wilks test. In all instances, the data were non-parametric, so only the Mann-Whitney U-test was utilized. A *p*-value < 0.05 was considered statistically significant.

## SUPPLEMENTARY MATERIALS FIGURES


