# Supplementary material for: Reversibility of glioma stem cells’ phenotypes explains their complex in vitro and in vivo behavior: Discovery of a novel neurosphere-specific enzyme, cGMP-dependent protein kinase 1, using the genomic landscape of human glioma stem cells as a discovery tool
Source: Oncotarget. 2016 Aug 24;7(39):63020–41. doi: 10.18632/oncotarget.11589 (PMC5325344; doi:10.18632/oncotarget.11589)
Supplement: Supplementary file 1 [file oncotarget-07-63020-s001.pdf]

Reversibility of glioma stem cells' phenotypes explains their complex *in vitro* and *in vivo* behavior. Discovery of a novel neurosphere-specific enzyme, cGMP-dependent protein kinase 1, using the genomic landscape of human glioma stem cells as a discovery tool

Supplementary Material

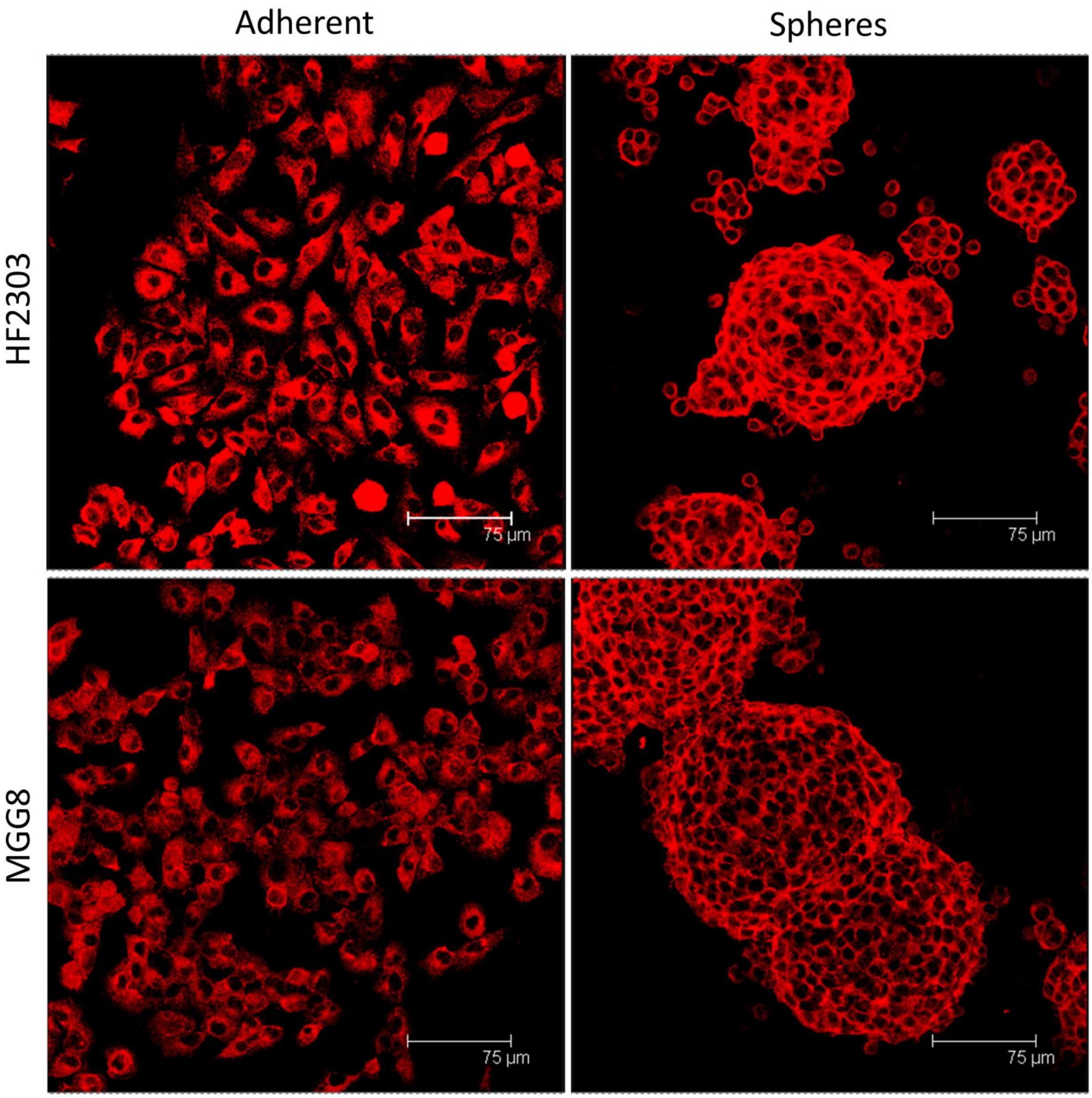

Figure S1. Immunostaining of human glioma stem cells HF2303 and MGG8 as adherent cultures and tumorspheres for CD133 are shown in this figure. Note that for both sets of human glioma stem cells immunoreactivity for CD133 is seen in both states, i.e., when growing as tumorspheres (enriched in glioma stem cells), or adherent (enriched in differentiated cells). As CD133 immunoreactivity did not distinguish between both states we could not utilize it as a differential marker for tumorspheres.

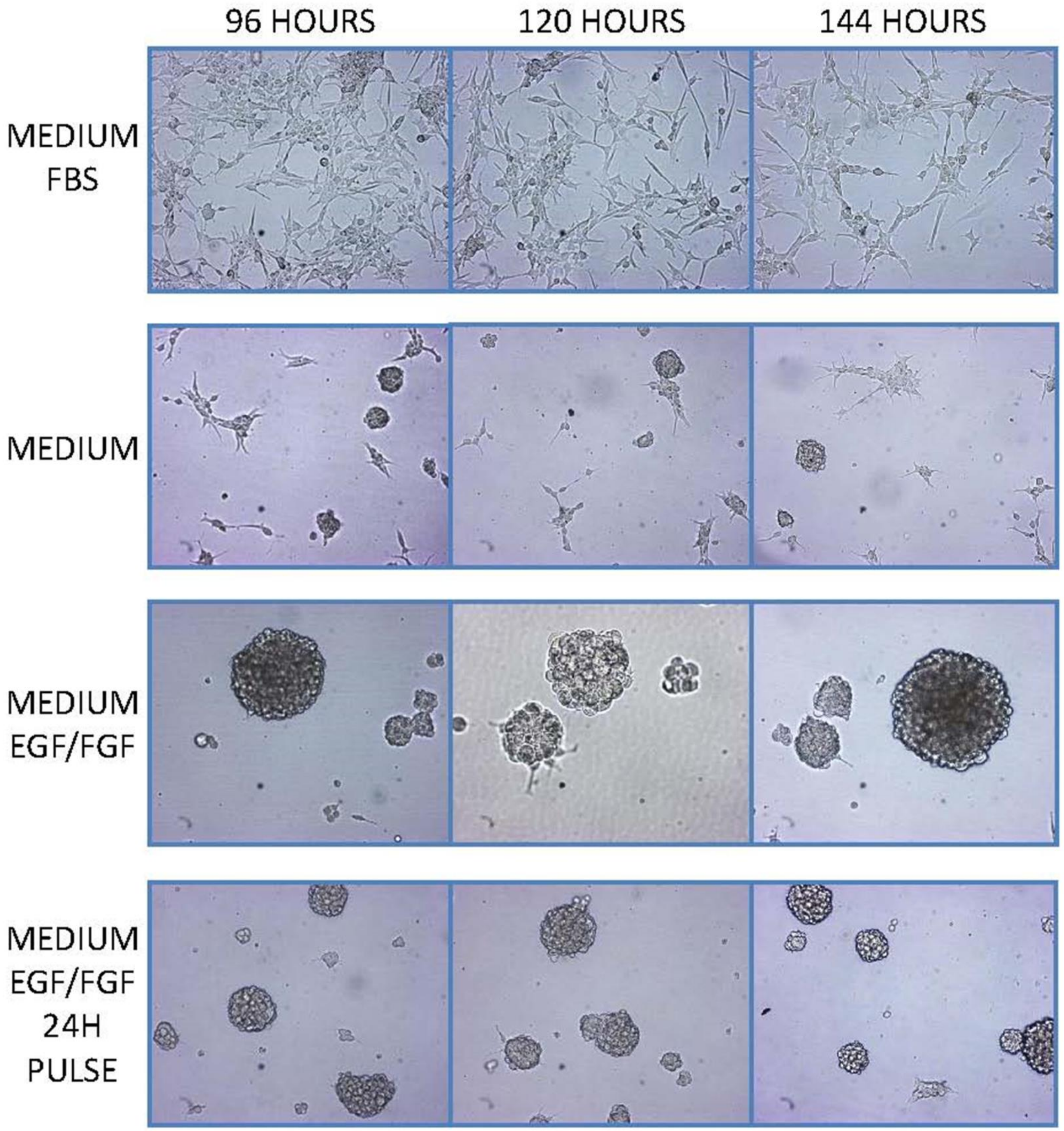

Figure S2. This figure illustrates the timing of neurosphere/tumorsphere formation in HF2303 cells. If adherent cells are incubated in medium + FBS no neurospheres/tumorspheres will form even after 144hrs of culture. If grown in medium alone only few small neurospheres/tumorspheres will form. Once EGF/FGF is added to the medium proper sized neurospheres/tumorspheres will

grow. These structures are fully formed at 96hrs. The use of a shorter EGF/FGF 24hrs pulse induces the formation of neurospheres/tumorspheres of smaller size. This suggests that continuous availability of EGF/FGF is needed for full neurosphere/tumorsphere development.
